# Synthetic α-Conotoxin Mutants as Probes for Studying Nicotinic Acetylcholine Receptors and in the Development of Novel Drug Leads

**DOI:** 10.3390/toxins2061471

**Published:** 2010-06-14

**Authors:** Christopher J. Armishaw

**Affiliations:** Torrey Pines Institute for Molecular Studies, 11350 SW Village Pkwy, Port St Lucie, FL 34987, USA; Email: carmishaw@tpims.org; Tel.: +1-772-345-4720; Fax: +1-772-345-3649

**Keywords:** α-conotoxin, nicotinic acetylcholine receptor, acetylcholine binding protein, structure-activity relationship studies, mutational analysis

## Abstract

α-Conotoxins are peptide neurotoxins isolated from venomous marine cone snails that are potent and selective antagonists for different subtypes of nicotinic acetylcholine receptors (nAChRs). As such, they are valuable probes for dissecting the role that nAChRs play in nervous system function. In recent years, extensive insight into the binding mechanisms of α-conotoxins with nAChRs at the molecular level has aided in the design of synthetic analogs with improved pharmacological properties. This review examines the structure-activity relationship studies involving α-conotoxins as research tools for studying nAChRs in the central and peripheral nervous systems and their use towards the development of novel therapeutics.

## Abbreviations

*Ac*: *Aplysia californica*
AChBP: acetylcholine binding protein
*Bt*: *Bulinus truncatus*
GABA: γ-aminobutyric acid
HEPES: (4-(2-hydroxyethyl)-1-piperazineethanesulfonic acid)
Laa: lipidic amino acid
*Ls*: *Lymnaea stagnalis*
nAChR: nicotinic acetylcholine receptor
NET: norepinephrine transporter
PS-SCL: positional scan synthetic combinatorial library
SCAL: safety catch amide linker
Sec: selenocysteine
SPPS: solid-phase peptide synthesis

## 1. Introduction

Since the pioneering work of Endean and colleagues, venoms from marine cone snails that inhabit tropical reef ecosystems have fascinated researchers due to their potent paralytic properties [1]. Given their relatively low mobility compared to other aquatic organisms, cone snails have evolved an efficient biological strategy to rapidly immobilize their prey. Their venom is injected via an elaborate harpoon mechanism, which utilizes a disposable spear-like radular tooth attached to a retractable thread loaded with toxic venom such that upon contact, its prey is immediately subdued [2]. The rapid paralysis effected by cone snail venom is the result of a highly complex mixture of disulfide rich peptide neurotoxins, known as conotoxins [3]. The first formal characterization of conotoxins by Olivera and co-workers in the 1980s attracted intense interest among neuroscientists and pharmacologists studying nervous system functions, by providing unique molecular probes and novel drug leads [4,5]. 

In contrast to many other known marine natural products, which are complex organic compounds produced by the action of enzymes, conotoxins are peptides that are expressed as genetically encoded combinatorial libraries [6]. Conotoxin genes are active in the venom ducts of cone snails, where they are translated as a larger precursor peptide, which undergoes posttranslational processing to produce the mature active conotoxin [7]. Across the approximately 500 known cone snail species, it has been estimated that there are more than 100,000 individual conotoxins with unique pharmacological properties [8]. Despite the complexity of cone snail venom, conotoxins have evolved from relatively few structural frameworks. Multiple disulfide bonds give rise to a series of intervening loops of amino acids, which contain a high degree of variability as a result of extensive mutation (*i.e.*, hypermutation). Some of the major classes include ω-conotoxins (voltage gated calcium channels), δ- and μ-conotoxins (voltage gated sodium channels), χ-conotoxins (norepinephrine transporter), ρ-conotoxins (α1A-adrenoreceptor), and α-conotoxins (nicotinic acetylcholine receptors) [9]. The diversity of ion-channels and receptors targeted by conotoxins makes them particularly useful research tools for studying the roles these receptors play in the central and peripheral nervous systems. Moreover, the therapeutic potential of conotoxins has been exemplified though the development of the calcium channel blocker Prialt^®^ (ω-Conotoxin MVIIA), an N-type calcium inhibitor that is used as an intrathecal analgesic for the treatment of chronic neuropathic pain [10].

## 2. α-Conotoxins as Probes for Nicotinic Acetylcholine Receptors

α-Conotoxins are competitive antagonists of nicotinic acetylcholine receptors (nAChRs) [11]. Nicotinic acetylcholine receptors belong to the superfamily of Cys-loop ligand-gated ion channels, which also includes 5-hydroxytryptamine, γ-aminobutyric acid (GABA), and glycine receptors [12]. Dysfunction of nAChRs is implicated in several neuropathological conditions, including cognitive dysfunction, neuropathic pain, and nicotine reward mechanisms [13]. 

Postsynaptic nAChRs are crucial mediators of the fast excitatory cholinergic neurotransmission in the central and peripheral nervous systems, which also influences the activity in several other important neurotransmitter systems, including dopamine, glutamate, and GABA [14,15]. All nAChRs bind the neurotransmitter acetylcholine, which induces channel opening through an allosteric mechanism [16]. Structurally, nAChRs are pentameric complexes composed of combinations of closely related α1–10, β1–4, δ, and ε/γ subunits, each consisting of an extracellular ligand-binding domain, four transmembrane helices, and an extended intracellular region, symmetrically arranged around a central cation conducting pore. Muscle-type nAChRs exist at the skeletal neuromuscular junction, and are composed of two α1-subunits and β1-, δ-, and γ/ε-subunits (αβδγ/ε) [17]. Neuronal nAChRs are either heteromeric combinations of α2–6 and β2–4-subunits, α9α10 complexes, or homomeric complexes consisting exclusively of α7 or α9 subunits [17]. The large number of different combinations of neuronal subunits gives rise to a large number of nAChR subtypes, each of which exhibits distinct neuropharmacological properties [18]. The different subtypes of nAChR subtypes are involved in a range of neuropathological conditions, including pain, nicotine addiction, autism, epilepsy, schizophrenia, Tourette’s syndrome, Alzheimer’s, and Parkinson’s diseases. As such, subtype specific ligands are profoundly important for studying the role that nAChRs play in such diseases to develop more effective therapeutic agents, with fewer side effects, than present options.

The development of small molecule agonists based on the structures of nicotine, epibatidine, and cytisine have been the subject of numerous drug discovery research programs for developing therapeutics that target nAChRs (for review, see Jensen *et al.* [14] and Arneric *et al.* [19]). However, issues regarding receptor subtype selectivity remain a significant challenge. For example, Varenicline (Chantix^TM^, Pfizer), which acts as a partial α4β2 nAChR agonist, was approved by the FDA in 2006 to treat nicotine withdrawal symptoms [20]. Varenicline has been shown non-selective for the α4β2 nAChR and has been shown to be a full agonist for the α7 subtype [21]. Furthermore, recent reports indicate that Varenicline may be associated with several adverse neuropsychiatric side effects, including depression and suicidal behavior [22,23]. 

On the other hand, α-conotoxins exhibit an exquisite ability to distinguish between different subtypes of nAChRs [9]. They are competitive antagonists of nAChRs that bind at the interface between α-subunits and β-subunits in heteromeric receptors, and between two α-subunits in homomeric receptors [17]. Their relative ease of chemical synthesis makes α-conotoxins useful for probing nAChRs in the central and peripheral nervous system, with promising therapeutic potential for treating pain and other conditions [24]. Nonetheless, unlike most small molecule candidates, issues concerning the administration of conotoxins that limit their general applicability as drugs need to be addressed.

**Figure 1:**
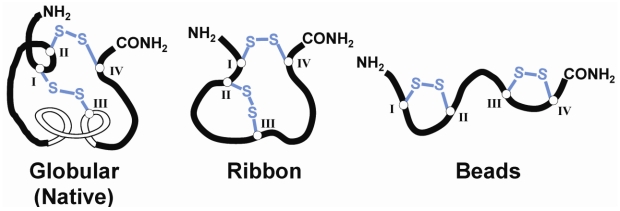
Schematic diagram representing the three possible disulfide bond isomers of α-conotoxins.

Typically, α-conotoxins consist of between 12–20 amino acid residues and contain two highly conserved disulfide bonds (Table 1). In native α-conotoxins, the disulfide bonds are connected in a (Cys^I^-Cys^III^),(Cys^II^-Cys^IV^) (globular) arrangement. Additional non-native isomers are also possible, namely the (Cys^I^-Cys^IV^),(Cys^II^-Cys^III^) (ribbon) and (Cys^I^-Cys^II^),(Cys^III^-Cys^IV^) (beads) isomers (Figure 1). The first and second cysteine residues are always adjacent, but the number of amino acid residues between the second and third cysteine, and between the third and fourth cysteine residues can vary. This gives rise to two loops of intervening amino acids denoted *m* and *n*, respectively. The cysteine framework refers to the number of residues in the *m* and *n* loops. For example, α-conotoxins with a 4/7 cysteine framework contain four and seven residues in their respective *m* and *n* loops. In addition to the intra-cysteine loops, some α-conotoxins, including EI [25], GID [26], ArIA [27], SrIA [28], and PIA [29], have an extended *N*-terminal region which contains amino acids that are also important for activity.

α-Conotoxins are among the most ubiquitous class of conotoxins identified so far, and the venom of nearly all *Conus* species is likely to contain at least one of these [30]. Furthermore, the number of α-conotoxins that are being characterized is rapidly increasing, as new isolation techniques become available [31,32]. Interestingly, identical conotoxins have been identified from different cone snail species. For example, Lp1.1, first identified from cDNA libraries of *Conus leopardus* venom [33], has also been independently characterized from the venom of *Conus litteratus* (designated as LtIA) [34]. 

Two sub-classes of α-conotoxins are able to discriminate between muscle and neuronal type nAChRs [35]. Thus far, α-conotoxins that target muscle nAChRs are predominantly found in fish hunting species of cone snails [36], and additional sequences continue to be identified from cDNA libraries [37]. While muscle specific α-conotoxins generally exhibit a 3/5 cysteine framework, α-conotoxins EI and PIB are exceptions, exhibiting 4/7 and 4/4 frameworks, respectively [25,38]. α-Conotoxins GI, GIA, and GII were the first conotoxins to be biochemically characterized [5], and their features are consistent in all other muscle specific α-conotoxins characterized to date, reflecting extensive homology among this class [39,40,41,42,43]. Although α-conotoxin SII contains one additional disulfide bond outside of the regular cysteine framework, its overall sequence and loop structure are consistent with other α-conotoxins [42]. 

The second sub-class of α-conotoxins has high specificity for neuronal nAChRs, and are among the most ubiquitous nAChR antagonists present in the venoms of fish, mollusk, and worm hunting cone snails [30]. Although more than half of the known α-conotoxins discovered to date exhibit a 4/7-cysteine framework, other neuronal α-conotoxins possessing unique cysteine frameworks are continually being discovered, including 4/6 (AuIB) [44], 4/4 (BuIA) [45], and 4/3 (ImI, ImII and RgIA) [46,47,48]. Additionally, several α-conotoxins possessing a 4/5 cysteine framework, including Ca1.1 and Pu1.3, have been identified from cDNA libraries [49,50]. Despite the occurrence of different cysteine frameworks in nature, systematic truncation of the *n*-loop in synthetic analogs of α-4/7-conotoxins leads to significantly decreased conformational stability and pharmacological activity [51].

Although their three-dimensional conformations are highly conserved, extensive mutation occurs within the α-conotoxin *m* and *n* loops and small differences in amino acid side chains can lead to profound changes in receptor subtype specificity [11,52]. With a few exceptions, nearly all neuronal α-conotoxins contain a conserved serine and proline residue in the *m*-loop (See Table 1). While not as prolific in α-conotoxins as in some other conotoxin classes, posttranslational modifications have been observed. Most α-conotoxins exist as *C*-terminal carboxamides, although some exceptions, including SII and GID, exhibit a *C*-terminal carboxylate [26,42]. Other posttranslational modifications include carboxylation of glutamic acid to γ-carboxyglutamic acid and hydroxylation of proline [26,53,54]. Sulfonation of tyrosine has been observed in several α-conotoxins, namely EpI, PnIA, PnIB, AnIA, AnIB, and AnIC [55,56,57,58]. Whereas incorporation of unsulfated tyrosine into α-conotoxins PnIA and PnIA does not appear to significantly affect activity [56], the unsulfated forms of EpI, AnIA, and AnIA display moderate decreases in antagonist potency [57,59].

## 3. Structural Studies of α-Conotoxins

**Figure 2:**
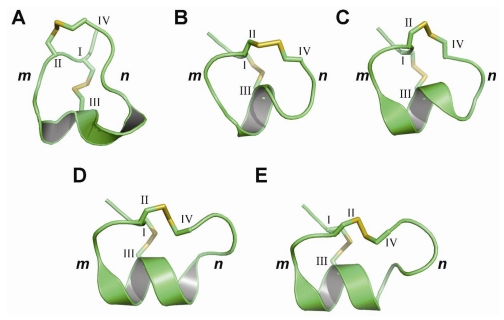
Three-dimensional structures of selected α-conotoxins representing five different cysteine frameworks. (**A**) X-ray crystal structure of α3/5-GI [61]; (**B**) NMR solution structure of α4/3-ImI [66]; (**C**) NMR solution structure of α4/4- BuIA [67]; (**D**) NMR solution structure of α4/6-AuIB [68]; (**E**) X-ray crystal structure of α4/7-PnIA [62]. Cysteine numbers and loop designations are indicated.

Three-dimensional structural studies provide insight into the role of specific residues involved in nAChR binding and biological activity (Figure 2) [60]. Due to their comparatively small size and the associated difficulties in crystallizing α-conotoxins, relatively few X-ray crystal structures of α-conotoxins have been reported in the literature [61,62,63]. As such, NMR spectroscopy is usually the method of choice for calculating three-dimensional structural studies of α-conotoxins and has been extensively conducted for many known α-conotoxins and their synthetic analogs [64]. Nonetheless, three-dimensional structures derived using both methods have been shown to exhibit very similar conformations. Moreover, NMR structures acquired independently by different research groups under varying conditions, including differing solvent environments, appear to be in good agreement [65].

**Table 1:** α-Conotoxins sequence alignment and their selectivity for nAChR subtypes. Conserved cysteine residues are shaded in grey. For all α-conotoxins, disulfide connectivity is between Cys^I^-Cys^III^ and Cys^II^-Cys^IV^. The conserved proline (or hydroxyproline) is boxed. Posttranslational modifications are defined as Z: pyroglutamate; Ø: Hydroxyproline; Ě: γ-carboxyglutamate; Ÿ: sulfated tyrosine; *: *C*-terminal amide; ^: *C*-terminal carboxylate.

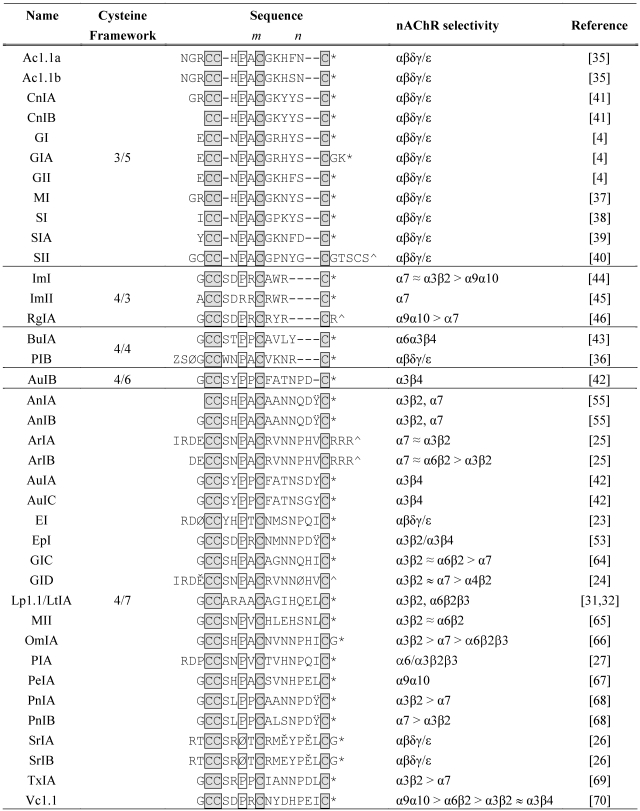

Despite their relatively small size, α-conotoxins adopt very well defined three-dimensional structures in solution that are stabilized by internal disulfide bonds, which are buried deep within the core of the molecule. They display a short 3_10_ helical segment braced by the disulfide bond between Cys^I^ and Cys^III^ that comprises the active portion of the molecule (Figure 2). A comparison of α-conotoxins with different numbers of residues in the *m*-loop shows that their structures overlay across the *n*-loop [60]. However, there is often a clear difference in the conformation of the *n*-loop region, even among conotoxins possessing the same number of residues in this loop, suggesting the impact of hypervariablity on the structure and function of α-conotoxins [60].

Another important conserved structural feature of α-conotoxins is the presence of a proline residue in the *m*-loop, which exists in the *trans* conformation and is responsible for inducing the 3_10_ helical turn motif that orients solvent exposed residues towards the nAChR binding site [65]. As such, substitutions of proline with other α-amino acids result in dramatic losses in nAChR activity, which can be attributed to a decrease in structural definition [47,76,77,78,79,80]. Nonetheless, novel conotoxins have been discovered that do not include a conserved proline, including α-conotoxins ImII [47] and Lp1.1/LtIA [33,34].

Misfolded disulfide bond isomers generally exhibit different 3D-conformations compared to native conotoxins, with the ribbon and beads isomers exhibiting greater conformational flexibility, which often results in lower pharmacological activity [60,81]. However, the non-native ribbon isomer of α-4/6-AuIB has been shown to exhibit 10-times more activity than the native conotoxin, suggesting a new level of conotoxin diversity for performing structure-activity relationship studies [82]. Interestingly, the globular isomer of α-4/4-BuIA demonstrates multiple conformations in solution, including conformers distinct from the native α-conotoxin folding motif [67].

While NMR spectroscopy has provided much valuable structural insight into α-conotoxins, an understanding of the structural basis of α-conotoxin binding to nAChRs has increased considerably in recent years with the published X-ray co-crystal structures of acetylcholine-binding proteins (AChBPs) in complex with various α-conotoxin ligands [83,84,85]. AChBPs are water soluble proteins isolated from various aquatic snails, and X-ray crystal structures of AChBPs from *Lymnaea stagnalis* (*Ls*-AChBP), *Aplysia californica* (*Ac*-AChBP), and *Bulinus truncatus* (*Bt*-AChBP) have been reported [86,87,88]. These proteins are expressed in molluskan glial cells and it has been proposed that their function is to modulate synaptic acetylcholine transmission [89]. AChBPs display sequence homology with the *N*-terminal ligand binding domain of several Cys-loop ligand-gated ion-channels, including nAChRs [12]. Moreover, they assemble into stable pentameric complexes characterized by binding affinities for nAChR ligands that are comparable to those exhibited by the homomeric α7 nAChR [90]. AChBPs are very useful structural surrogates of nAChRs and other classes of ligand-gated ion channels [91,92,93]. However, recently reported high-resolution X-ray crystal structures of ligand-gated ion channels promise to provide greater structural insight into nAChRs at the molecular level [94,95,96,97].

The relatively high degree of homology between nAChRs and AChBP provides the opportunity for computational modeling of α-conotoxin-receptor interactions [98]. Docking of α-conotoxins ImI, PnIA, PnIB, and MII into α7 and α3β2 nAChR homology models derived from AChBP crystal structures reveals insights into α-conotoxin binding modes at these receptors [99]. These studies indicate the ImI and PnIB binding site is located above the β9/β10 hairpin of the α7 nAChR subunit. Interestingly, PnIB, PnIA, and MII were found to bind in a similar location on α7 or α3β2 receptors, predominantly through hydrophobic interactions, while ImI bound further from the ACh binding pocket, mostly through electrostatic interactions. Other docking studies of RgIA have been reported using an α9α10 nAChR homology model derived from AChBP structures to reveal specific binding interactions [100]. 

**Figure 3:**
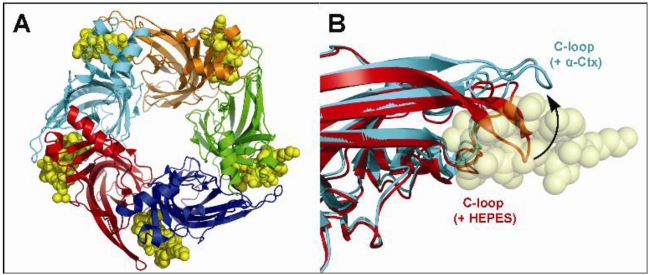
(**A**) Top view of the X-ray co-crystal structure of α-conotoxin PnIA[A10L,D14K] bound to *Ac*-AChBP (PDB ID: 2BR8) [83]. Each subunit is shown in a different color to highlight the pentameric arrangement of *Ac*-AChBP, and the bound α-conotoxin is shown in yellow; (**B**) Overlay of a single AChBP subunit with α-conotoxin PnIA[A10L,D14K] bound, showing an open *C*-loop (cyan), and with HEPES bound, showing a closed *C*-loop (red). The α-conotoxin in the open *C*-loop structure is shown in yellow (adapted from Celie *et al.* [85]).

X-ray co-crystal structures of α-conotoxins ImI and PnIA[A10L,D14K] bound to *Ac*-AChBP provide extensive detail into the binding interactions of α-conotoxins with nAChRs and has allowed for the construction of more reliable homology models [83,84,85]. These structures show that upon binding, α-conotoxins are buried deep within the ligand binding site and interact with residues on both faces of adjacent subunits, with the toxin occupying all five binding sites of AChBP (Figure 3A) [85]. The toxin also opens the *C*-loop of AChBP and induces a rigid-body subunit movement (Figure 3B). Interestingly, AChBP does not induce any structural changes in the bound conotoxin, with X-ray crystal structures of both free PnIA and bound PnIA[A10L,D14K] overlapping, suggesting that the α-conotoxin structural framework is rigid, and binding is solely determined by the ability of the receptor to adapt to the conotoxin [85]. Another method for studying α-conotoxin/ AChBP interactions involves saturation transfer difference NMR, which was been used to study complexes of Vc1.1 and MII bound to *Ls*-AChBP [101]. This study broadly highlights the utility of this approach by showing that aromatic residues present on the helical barrel of these α-conotoxins (Tyr^10^ of Vc1.1 and His^12^ of MII) display strong interactions deep within the nicotinic binding site.

AChBPs have also proven to be useful tools in the discovery of new α-conotoxins from cone snail venom extracts, with the discovery of α-conotoxins OmIA and TxIA using AChBP competition binding assays [71,74]. Interestingly, nAChR ligands have been shown to display different binding affinities for AChBPs from different species. For instance, α-conotoxin ImI exhibits a 16,000-fold greater affinity for the *Ac*-AChBP over *Ls*-AChBP [87], while OmIA shows similar binding affinity for AChBP isolated from all three species [71]. Similarly, substitution of Asp^14^ with Lysine in PnIA[A10L] (PnIA[A10L,D14K]) resulted in an analog with high affinity for *Ac*-AChBP and *Ls*-AChBP [85]. 

## 4. Synthetic Mutants of α-Conotoxins

**Figure 4:**
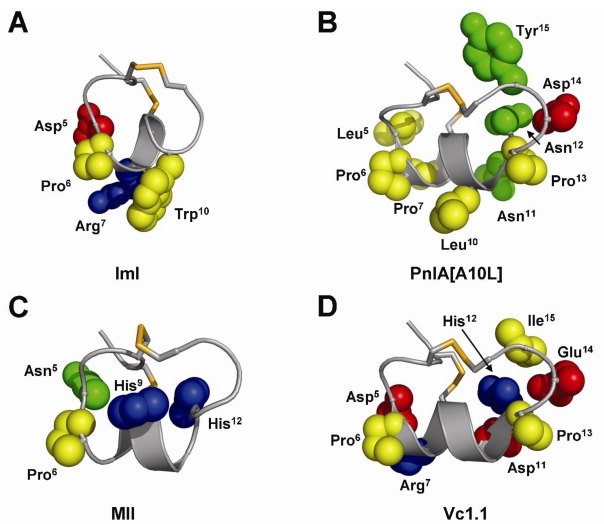
Summary of pharmacophore residues of selected α-conotoxins, as determined by mutagenesis studies. Nonpolar residues are shown in yellow; polar uncharged, green; acidic, red; basic, blue.

Mutational analysis of the hypervariable regions of α-conotoxins allow useful structure-activity relationships to be elucidated. As such, alanine scanning mutagenesis, as well as systematic replacement with other amino acid residues, allows one to determine the importance of function at each position. Generally, a significant change in activity for a mutated residue provides information on the importance of a given position in the conotoxin sequence. Such studies can reveal a great deal of information regarding pharmacophoric interactions of conotoxins with nAChRs (Figure 4).

### 4.1. α-Conotoxins ImI and ImII

α-Conotoxin ImI, from the worm hunting cone snail *Conus imperialis*, was the first neuronal α-conotoxin to be discovered that displaced the α7 nAChR selective snake toxin α-bungarotoxin [46], however it was later shown that the toxin is also active at the α3β2 subtype, and weakly active at the α9α10 subtype [102]. Nonetheless, given its relatively high selectivity and ease of chemical synthesis, ImI has been the subject for numerous structure-activity relationship studies. Alanine scanning of ImI indicated that Arg^7^ plays a major role in ImI α7 nAChR binding, with a significant decrease in activity observed for the ImI[R7A] analog (Figure 4a) [103]. Similarly, ImI[P6A] exhibited a profound decrease in activity, where this observation can be attributed to the loss of structural definition brought about by the conserved, conformationally restricted proline residue. Extensive NMR structural studies reveal that minor conformational changes of ImI mutants can result in significantly reduced pharmacological activity [104,105].

Several point mutations in ImI revealed its binding determinants to the α7 nAChR. Substitution of the Arg^7^ position with lysine was investigated, and despite maintaining the positive charge, resulted in significant losses in activity [76]. Similarly, substitution of Arg^7^ with glutamine or glutamic acid also resulted in analogs with significantly lower activity, underlining the importance of this residue as a crucial determinant for ImI binding to α7 nAChR [76]. While Arg^7^ was originally believed to form important π-cation interaction with conserved aromatic residues in the principle binding site of the receptor [99], it was later suggested, through homology modeling, that the positive charge of Arg^7^ is stabilized by an intramolecular salt bridge in ImI, and van der Waals interactions with the receptor binding site [85]. Substitution of Trp^10^ with other residues, including phenylalanine and tyrosine, resulted in analogs with similar activity to WT-ImI [76,106], indicating the importance of an aromatic side-chain residue at this position.

**Figure 5:**
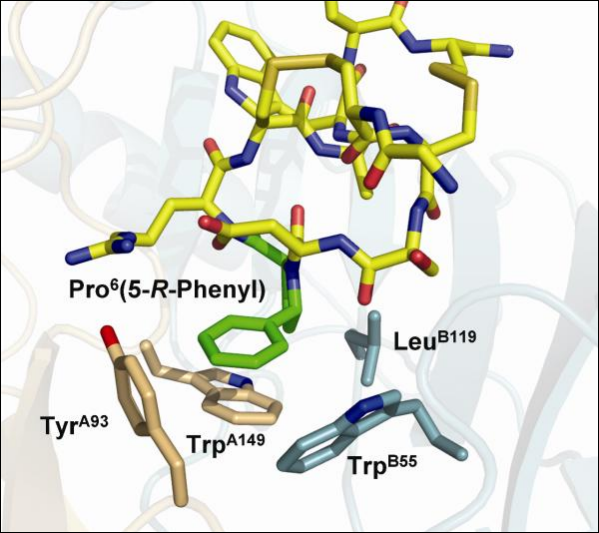
Homology model of α-conotoxin ImI-[Pro^6^(5-*R*-phenyl)] bound to the α7 nAChR. Residues that form the binding pocket for endogenous ligands are indicated. The 5-*R*-phenylproline residue is highlighted in green and each of the two subunits is indicated in orange and cyan, respectively. Adapted from Armishaw *et al.* [107].

Close inspection of X-ray co-crystal structures of α-conotoxin ImI bound to *Ac*-AChBP reveals that the conserved proline in these conotoxins is oriented towards the binding pocket of endogenous ligands [83,84]. However, the conserved proline does not take part in any ligand/receptor interactions. Given that the conserved proline is important for maintaining the three-dimensional conformation of α-conotoxins, a series of α-conotoxin ImI derivatives were synthesized that incorporated substituted proline derivatives in position 6, resulting in several analogs with increased activity for the α7 nAChR [107]. An α7 nAChR homology model derived from *Ac*-AChBP reveals that a phenyl substituent in the 5-*R*-position of Pro^6^ in ImI leads to an efficient π-stacking interaction with the binding site residues (Figure 5). However, the same substitution in PnIA[A10L] significantly decreased activity at the α7 nAChR [107].

α-Conotoxin ImII, also isolated from *Conus imperialis*, is another potent α7 nAChR antagonist [47]. Unlike ImI, ImII does not compete with α-bungarotoxin binding, which suggests a different binding site on the α7 nAChR [102]. To characterize the binding mode of ImII in more detail, a series of ImII mutants were investigated by Tsetlin and co-workers, including ImII[W10Y] and the ribbon isomer of ImII [108]. Both isomers displaced [^125^I]-α-bungarotoxin from human α7 nAChRs, as well as in *Ac*-AChBP and *Ls*-AChBP. On *Torpedo* nAChR, radiolabeled [^125^I]-ImII[W10Y] revealed specific binding and was readily displaced by WT-ImII, ImII[W10Y], and the ImII ribbon isomer [108]. However, a higher concentration of ImI was required to displace [^125^I]-ImII[W10Y], thus providing further evidence for a distinct binding site for ImII.

### 4.2. α-Conotoxins PnIA, PnIA and TxIA

Two peptides isolated from the venom of the molluscivorous cone snail, *Conus pennaceus*, are α-conotoxins PnIA and PnIB. Although they display very similar amino acid sequences, both were shown to target different nAChR subtypes (see Table 1) [73]. Two chimeric analogs were synthesized, resulting in a single amino acid substitution of PnIA at position 10 to leucine (PnIA[A10L]) and at position 11 to serine (PnIA[N11S]) to investigate the extent that each residue contributes to activity [109,110]. Interestingly, PnIA[A10L] demonstrated a complete switch in selectivity from α3β2 to α7 nAChRs, while PnIA[N11S] showed reduced activity at both subtypes. Further alanine scanning of PnIA[A10L] indicates that residues between at position 15, and those between positions 5 and 13, were important for activity at the α7 nAChR (Figure 4b) [111].

α-Conotoxin TxIA was discovered from crude venom extracts of *Conus textile* using an [^125^I]-α-bungarotoxin binding assay against *Ls*-AChBP [74]. TxIA was shown to have a higher affinity for *Ls*-AChBP than any previously identified α-conotoxin and is selective for α3β2 nAChRs over the α7 subtype. A comparison of the TxIA sequence with PnIA shows that these two conotoxins differ by only three residues. A series of TxIA mutants suggested that long chain hydrophobic residues at positions 9 or 10 were important for activity, leading to the to TxIA[A10L] analog, which showed a greater antagonistic potency for the α7 nAChR than WT-TxIA. An X-ray co-crystal structure of TxIA[A10L] with *Ac*-AChBP revealed a distinct binding orientation, with a 20° backbone tilt when compared to the other α-conotoxin/AChBP binding complexes [74]. Furthermore, these structural studies revealed an important salt bridge between Arg^5^ of TxIA[A10L] and Asp^195^ of AChBP.

### 4.3. α-Conotoxin MII

α-Conotoxin MII was the first neuronal α-conotoxin to be isolated from a fish hunting cone snail. Its features are typical of most other neuronal α-conotoxins [70]. MII was first found to selectively inhibit the α3β2 nAChR. However, later studies showed that it also blocks α6-containing nAChRs [112]. Alanine scanning allowed the identification of Asn^5^, Pro^6^, and His^12^ as major determinants for potency at the α3β2 nAChR and α6-containing subtypes (Figure 4c) [113]. The MII[E11A] analog was shown to increase selectivity for the α6β2 and α6α4β2 subtypes [114,115]. Comparisons of the NMR structure of MII[E11A] suggest an increased hydrophobic area, relative to other α-conotoxins, may be responsible for its selectivity for the α6β2 nAChR [116]. The novel MII[S4A,E11A,L15A] analog was synthesized and allowed for the identification of amino acid residues in nAChR subunits that confer selectivity for α3- and α6 subunits [117]. The discovery of α-conotoxin MII as an α6-subunit nAChR antagonist has led to the characterization of additional α-conotoxins that bind both α3β2 and α6β2 nAChRs, including GIC [69], BuIA [45], PIA [29], and OmIA [71]. The binding of OmIA to various AChBPs provides a unique opportunity for developing homology models of α6-containing nAChR subtypes.

### 4.4. α-Conotoxin GID

α-Conotoxin GID has been identified as an antagonist for the α4β2 nAChR, although this conotoxin also blocks the α7 and α3β2 nAChR subtypes with a higher degree of potency [26]. Unlike most other α-conotoxins, GID contains a *C*-terminal carboxylate, whereby substitution with a *C*-terminal carboxamide results in a loss of activity at the α4β2 nAChR [118]. Another non-typical feature is the presence of arginine at position 12, which is usually a hydrophobic or aromatic residue in other α-conotoxins. Given the therapeutic relevance of the α4β2 nAChR in pain and nicotine addiction [17], GID has been the subject of mutagenesis studies. The mutation of Arg^12^ to alanine leads to a significant decrease in activity at the α4β2 nAChR, but not the α3β2 and α7 nAChRs [26]. The Asn^14^ residue was also identified as being directly involved in interactions with the receptor [118]. 

Even more significant is the presence of an *N*-terminal tail consisting of four amino acid residues that contains a posttranslationally modified γ-carboxyglutamate (Gla) residue at position 3. While mutation of position 3 from Gla to glutamic acid in GID does not affect pharmacological activity, removal of the *N*-terminal tail leads to a significant decrease in antagonistic potency for the α4β2 nAChR [26]. However, the truncated peptide retains activity at α7 and α3β2 subtypes. This strongly suggests an important role for the *N*-terminal tail in receptor subtype selectivity. Despite this finding, an alanine scan of GID indicates that while all residues within the cysteine framework are necessary for binding to α3β2 and α7 nAChRs, specificity for the α4β2 subtype is not necessarily limited to the *N*-terminal tail, with Pro^9^ playing an important role in maintaining the three-dimensional conformation of GID, as well as the roles of Arg^12^ and Asn^14^ in receptor binding interactions.

### 4.5. α-Conotoxin ArIB

α-Conotoxin ArIB, which was isolated from cloning of *Conus arenatus* cDNA libraries, possesses the same extended *N*-terminal tail as GID [27]. Although the predicted sequence contains Glu at position 3, it is possible that this residue is modified to Gla in the mature toxin. Through reference to previous mutagenesis studies of MII and PnIA, a series of directed substitutions in ArIB led to the synthesis of ArIB[V11L,V16D], which was found to be highly selective for the α7 nAChR, and is the most selective ligand for this receptor reported to date [27]. Given the higher level of selectivity for the α7 nAChR when compared to [^125^I]-α-bungarotoxin, a radiolabeled [^125^I]-ArIB[V11L,V16D] analog was recently developed, and may find widespread use as a selective pharmacological probe [119].

### 4.6. α-Conotoxins RgIA and Vc1.1

Antagonists of the α9α10 nAChR are believed to be important targets for antinociceptive drugs that treat chronic neuropathic pain [120,121]. PeIA was the first α-conotoxin shown to preferentially block the α9α10 nAChR over the α7 nAChR [72]. Vc1.1 and RgIA show greater selectivity for the α9α10 nAChR than PeIA does [122,123,124,125]. Both of Vc1.1 and RgIA been shown to suppress a vascular response to pain in rats, which are involved in the transmission of pain [75]. While RgIA is the most selective α9α10 nAChR antagonist identified to date, Vc1.1 has also been found to block α6-containing subunits, as well as α3-containing nAChRs with lower potency [120,125]. Both Vc1.1 and RgIA target the α9α10 nAChR, although it has been proposed that Vc1.1 and RgIA also act as G-protein coupled GABA_B_ receptor agonists that modulate Ca_v_2.2 channels, resulting in their antinociceptive properties [126]. Vc1.1 has been shown to be an effective analgesic against pain in rat models following subcutaneous or intramuscular administration [122], and has been the subject for human clinical trials [24]. However, *in vitro* data indicated that Vc1.1 was ~100-fold less potent for human nAChRs compared to rat nAChRs, hence clinical development has been discontinued [124].

Intriguingly, Vc1.1 and RgIA both share the same *m*-loop sequence (SDPR) also found in α-conotoxins ImI and EpI, which are more selective for the α7 and α3β2 nAChR subtypes, respectively. As such, selectivity for the α9α10 nAChR for Vc1.1 and RgIA is likely to be attributed to residues in the *n*-loop. Scanning mutagenesis of Vc1.1 using alanine, aspartic acid, and lysine, identified residues important for activity at the α9α10 nAChR as being Asp^5^-Arg^7^ and Asp^11^-Ile^15^ (Figure 4d) [127]. Notably, several substitutions, in positions 4 and 9, were shown to be more potent at the α9α10 nAChR than WT-Vc1.1 was. A second generation of novel mutants was synthesized, leading to the identification of several analogs including Vc1.1[N9G], Vc1.1[N9I], Vc1.1[N9L], Vc1.1[S4R], and Vc1.1[S4K,N9A] that were more potent and selective for the α9α10 nAChR than WT-Vc1.1 was [127]. 

RgIA shares a very high degree of sequence homology with ImI, differing only in position 9 (Arg in RgIA; Ala in ImI) and position 10 (Tyr in RgIA; Trp in ImI), as well as the presence of an additional arginine at the *C*-terminal in RgIA (see Table 1). Side-chain mutagenesis of the RgIA *m*-loop, including Asp^5^, Pro^6^ and Arg^7^, were each shown to be crucial for inhibition of both the α9α10 and α7 subtypes [128]. Mutagenesis of the *n*-loop residues showed that RgIA[Y10W] exhibited near identical activity to the WT-RgIA, which was comparable to earlier mutagenesis studies involving ImI[W10Y], as discussed previously [76]. Similarly, the absence of the *C*-terminal arginine residue had no significant impact on activity. However, Arg^9^ in the *n*-loop of RgIA was shown to be critical for specific binding to the α9α10 subtype. This can be attributed to the positively charged arginine side chain that directly interacts with the α9α10 nAChR, since WT-RgIA and RgIA[R9A] both exhibit identical backbone NMR structures [128].

## 5. High Throughput Synthesis of α-Conotoxin Analogs

Preparation of α-conotoxins by solid-phase peptide synthesis (SPPS) provides a rapid and facile route to significant quantities of native and modified material for use in structure-activity relationship studies [129]. Despite their relative ease of synthesis and the vast number of novel α-conotoxin analogs prepared to date, most of these have been obtained using time and labor intensive low-throughput synthetic methodology. Furthermore, the selective formation of disulfide bond isomers in large arrays of α-conotoxin analogs remains a significant challenge [130]. While a large majority of mutagenesis studies of α-conotoxins to date have primarily focused on single amino acid substitutions to obtain structure-activity relationships, the large number of possible amino acid combinations in α-conotoxins makes the identification of active mutants “hit or miss”. As such, accelerated synthetic methodologies are required to rapidly identify α-conotoxin analogs that selectively inhibit nAChRs and other novel pharmacological targets.

A high throughput synthetic methodology that accelerates production of conotoxins has been proposed, which promises access to a larger number of analogs in a shorter time frame than previously achievable [131]. Central to this methodology is the use of the safety catch amide linker (SCAL) [132], which allows simultaneous removal of side chain protecting groups and linker activation, followed by liberating the peptide into solution by reductive amidolysis. Disulfide bonds are formed non-selectively directly from the cleavage mixture using DMSO oxidation, resulting in varying mixtures of disulfide bond isomers. A regioselective on-resin supported oxidation, using selenocysteine, was recently reported. This oxidation allows regioselective formation of disulfide bond isomers in a high-throughput fashion [133]. Although the use of SCAL methodologies has been demonstrated for the high throughput production of conotoxin analogs, its general applicability is limited by side reactions involving sensitive amino acid residues, particularly irreversible alkylation of tryptophan. Nonetheless, these methodologies will no doubt find useful applications in the high throughput production of α-conotoxins.

Mixture-based combinatorial methods are emerging for the high throughput production of α-conotoxins and their analogs [134]. It is well accepted that natural product extracts provide a valuable source of bioactive compounds with therapeutic relevance. Such extracts, including those from *Conus* venoms, are typically composed of thousands of different compounds in varying concentrations [135]. On the other hand, synthetic mixture-based combinatorial libraries are systematically arranged mixtures of compounds that contain every possible combination of the building blocks used in their synthesis [136]. Positional scan synthetic combinatorial libraries (PS-SCLs) provide a rapid means of acquiring functional information regarding all possible variable positions within a chemical framework [137]. As such, one can accurately deconvolute active sequences for a particular biological target from large mixtures of individual compounds that exist in very low concentrations in the assay sample. 

Although PS-SCLs have been used extensively by numerous researchers over the years for the successful discovery of high potency ligands for a wide range of biological targets [137,138,139,140,141,142,143,144,145], the use of this technique has been not been applied to conotoxins until recently. A synthetic combinatorial strategy for the high throughput production of α-conotoxin ImI analogs broadly highlights the utility of PS-SCLs in α-conotoxin structure-activity relationship studies, and allows for the design of potent and selective analogs [134]. Synthesis and pharmacological screening of a mixture based PS-SCL allowed amino acids that confer antagonistic activity to be identified. Significantly, three aromatic residues in position 10, tryptophan, tyrosine, and phenylalanine, were identified as being important for activity, which is consistent with results from previous structure-activity relationship studies [76,106]. Substitutions in position 9, including norleucine and leucine, as well as position 11, including histidine and tryptophan, were identified, prompting the synthesis of a second generation of individual α-conotoxin analogs, which provided several analogs exhibiting improved antagonistic potency for the α7 nAChR. A third generation of analogs was designed based on homology modeling studies using *Ac-*AChBP X-ray co-crystal structures to produce analogs with even greater antagonistic potencies by incorporation of other non-natural amino acid derivatives. A total of 96 individual ImI mutants were synthesized in high yield and purity, which is the largest number of reported α-conotoxin analogs produced in a single study to date [134]. A drawback of using synthetic α-conotoxin combinatorial libraries is the reliable folding of disulfide bonds to the native disulfide bond isomer in complex mixtures. In this regard, the development of new regioselective on-resin disulfide bond forming strategies may prove useful in overcoming this limitation [133].

Combinatorial strategies have profound potential for discovering α-conotoxins with novel pharmacological activities. For example, given the sequence similarities between ImI and RgIA, screening of the ImI *n*-loop PS-SCL for activity at the α9α10 nAChR could potentially lead to highly potent and specific antagonists for this receptor. However, at the present time, screening of the large number of samples generated in PS-SCLs is restricted to labor intensive electrophysiological recordings. Nonetheless, as more medium to high throughput screening assays become available for novel nAChR receptor subtypes, combinatorial libraries of α-conotoxins would be expected to be used more widely in structure-activity relationship studies and in the development of potent and specific nAChR ligands, as well as of ligands for other classes of ion channels and receptors.

## 6. Novel α-Conotoxin Analogs with Enhanced Pharmacokinetic Properties

Despite the therapeutic potential of α-conotoxins, the issue of *in vivo* stability and bioavailability remains a significant limitation. As with other classes of bioactive peptides, α-conotoxins generally exhibit poor *in vivo* stability, due to their susceptibility to degradation by endo- and exoproteases. Furthermore, issues regarding bioavailability and membrane permeability limit the general applicability of conotoxins as drugs. As such, much effort has focused on improving the pharmacokinetic properties of α-conotoxins while maintaining their potency and selectivity for nAChR subtypes.

It is well known that *N*-to-*C* cyclic peptides exhibit improved stability *in vivo* over linear peptides and have more restricted conformations [146]. Given the potential of α-conotoxins as *in vivo* research tools and drug leads, a valuable approach to improving their physical stability is to link their *N*- and *C*- termini. This approach has been successfully investigated and used for preparing stable and potent analogs of α-conotoxin MII [147]. Importantly, the spatial relationship between the *N*- and *C*-termini must be maintained, hence an oligopeptide spacer unit is required to preserve the three-dimensional conformation of the native α-conotoxin. When an appropriate spacer length was utilized, cyclic analogs of MII were shown to exhibit greatly improved stability over the native peptides, yet their three-dimensional structure and pharmacological activity were retained. This strategy has also been successfully applied to the synthesis of cyclic χ-conotoxin MrIA analogs [148]. Recently, it was reported that cyclization of α-conotoxin ImI led to the preferential formation of the ribbon disulfide bond isomer, particularly when a shorter oligopeptide spacer length was selected [149]. As such, regioselective disulfide bond formation was required to obtain the native globular isomer, and it was shown that cyclic globular analogs of ImI exhibited superior stability compared to cyclic ribbon analogs, which demonstrated comparable stability to WT-ImI.

Peptides, in general, do not pass easily though biological membranes, such as the gastrointestinal tract and the blood-brain barrier. As such, the issues of membrane permeability and oral availability have also been explored. Incorporation of a lipidic amino acid (Laa), 2-amino-d,l-dodecanoic acid into MII at the *N*-terminal, as well as substitution of Asn at position 5 was shown to significantly improve permeability across Caco-2 cell monolayers for both analogs, while maintaining inhibitory potency at the α3β2 nAChR [150]. Furthermore, NMR analysis revealed both Laa-MII analogs possessed a similar structure to WT-MII. An *in vivo* biodistribution study following oral administration of Laa-MII analogs in rats showed that although uptake was not significantly enhanced, the compounds did pass through the gastrointestinal tract, as suggested by increased accumulation of the compounds in the liver [151]. However, neither Laa-MII analog crossed the blood-brain barrier, underlining the importance of further investigation into developing novel α-conotoxin analogs that can permeate biological membranes. A cyclic χ-conotoxin MrIA has been reported where Laa’s were attached to the oligopeptide spacer unit and exhibited comparable activity to WT-MrIA [152], although biodistribution studies of these analogs are yet to be performed to assess their permeability across the blood-brain barrier.

**Figure 6:**
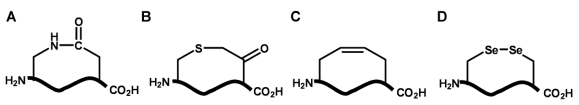
Non reducible disulfide mimetics that have been incorporated into α-conotoxin analogs. (a) lactam; (b) thioether; (c) dicarba; and (d) diselenide bridges.

Another concern with α-conotoxins as drugs is that their disulfide bond frameworks are susceptible to scrambling to other isomers under physiological conditions. To address this issue, replacement of the disulfide bond framework with non-reducible disulfide mimetics has been investigated by several groups (Figure 6). Lactam bridges were initially investigated by Barany and coworkers using α-conotoxin SI as a model (Figure 6a) [153]. Systematic replacement of the Cys^2^-Cys^7^ disulfide bond with a lactam bridge in two orientations resulted in complete loss of activity at muscle type nAChRs. However, replacement of the Cys^3^-Cys^13^ disulfide bond resulted in ~70-fold increase in affinity for one lactam orientation. A synthetic analog of α-conotoxin GI that substituted both disulfide bonds with a thioether mimetic has been investigated to improve the stability of conotoxins (Figure 6b). However, these analogs resulted in profound decreases in pharmacological activity [154]. The changes in activity for both studies can be directly attributed to differences in the bond geometry between disulfide bonds and these mimetics.

Dicarba-linkages more closely resemble the bond geometry of disulfide bonds, and have been successfully incorporated in α-conotoxin ImI, resulting in analogs with improved stability (Figure 6c) [155]. Cysteine residues at positions 2 and 8 were substituted with allylglycine, followed by on-resin microwave assisted ring-closing metathesis. The dicarba-ImI analog was shown to exhibit very similar antagonistic properties α7 nAChRs compared to WT-ImI in two different functional assays. Structurally, the NMR solution structure of the dicarba-ImI analog was very similar to the reported structure for WT-ImI. Nonetheless, minor structural differences were attributed to the different covalent geometry of the dicarba moiety compared to a disulfide bond, since the carbon-carbon double bond is significantly shorter than a corresponding sulfur-sulfur bond.

A more conservative disulfide bond isostere, the diselenide bond, has been shown to enhance disulfide bond stability under reducing conditions and is a convenient folding tool for synthesizing α-conotoxins (Figure 6d) [133,156], as well as other more complex conotoxin frameworks [157,158]. Selenocysteine (Sec) is a naturally occurring amino acid, which forms an essential catalytic group in several redox enzymes. It exhibits the propensity to oxidatively form a diselenide bond analogous to the disulfide bond and exhibits very similar bond geometry [159]. However, selenocysteine has a higher oxidation potential than cysteine, which allows it to be selectively oxidized over cysteine under very mild conditions [160]. 

A series of α-conotoxin ImI analogs, termed “α-selenoconotoxins” were synthesized by solid phase peptide synthesis with complementary replacement of either one ([Sec2,8]-ImI or [Sec3,12]-ImI), or both ([Sec2,3,8,12]-ImI) disulfide bonds with diselenide bonds [156]. Each analog demonstrated remarkable stability to reduction or scrambling under a range of chemical and biological reducing conditions, such as blood plasma thiols. Three-dimensional structural characterization by NMR and CD spectroscopy indicated conformational preferences that were very similar to native ImI, suggesting fully isomorphic structures. Additionally, full bioactivity was retained at the α7 nAChR, with each α-selenoconotoxin exhibiting a dose response curve that overlaps with WT-ImI. This work demonstrated that selenoconotoxins can be used as highly stable scaffolds for the design of new conotoxin based drugs. Recently, α-conotoxins representing five different cysteine frameworks were synthesized using SCAL methodology, demonstrating exclusive formation of the native disulfide bond isomers in all cases [133]. As was shown in previous studies, the α-selenoconotoxins exhibited similar antagonist potency for nAChR subtypes, with improved stability in human blood plasma. Furthermore, the X-ray crystal structure of α-selenoconotoxin PnIA demonstrated a fully conserved fold when compared to native PnIA. These studies highlight the utility of selenocysteine technology to high throughput α-conotoxin synthesis, since successive isolation steps are not required following cleavage.

## 7. Conclusions and Future Perspectives

The chemical synthesis of α-conotoxins for use in structure-activity relationship studies has led to the development of novel analogs that can be used as valuable research tools for studying the roles that nAChRs play in various neuropathological disorders and disease states. Furthermore, X-ray crystal structures of α-conotoxin/acetylcholine binding protein complexes permit more accurate homology models of nAChRs to be developed, allowing for the rational design of novel analogs with refined pharmacological properties. However, high-throughput synthetic methods and combinatorial strategies promise to greatly accelerate the identification of α-conotoxin analogs that are selective for nAChR subtypes, and other novel pharmacological targets. Despite their promising therapeutic potential, improving the pharmacokinetic properties of α-conotoxins remains an issue that needs to be addressed. Nevertheless, new strategies for improving the *in vivo* stability and membrane permeability of α-conotoxins continue to be investigated by various research groups toward the development of α-conotoxins as novel therapeutics. 
